# Syphilitic Affections of the Nervous System*A Thesis recommended for publication by the Faculty of the Buffalo Medical College.

**Published:** 1874-10

**Authors:** Edward N. Brush

**Affiliations:** Buffalo


BUFFALO

### Medical and Surgical Journal.

VOL XIV. OCTOBER, 1874. No. 3.

### Original Communications.

———:o:———

ART. I.—*Syphilitic Affections of the Nervous System.** *By* EDWARD N. BRUSH, M. D.

The comparative frequency of Nervous Disorders in Syphilitic patients, and the thorough consideration which they have received in the recent treatises upon Venereal Diseases, and especially in those devoted to a consideration of the nervous lesions in syphilis, would seem to indicate that he who attempted to enlarge still further upon the subject, was anticipating a want which did not exist in the literature of the medical profession.

Syphilitic disorders of the nervous system are of frequent occurrence; yet this fact, and the fact that as a rule the result of proper treatment in these cases is highly satisfactory, seems to attract but little attention from the majority of practitioners.

What physician is there, when called upon to treat certain diseases known to be influenced by a peculiar diathesis to which the patient is subject, whether it be gouty, rheumatic, tubercular or scrofulous, who is not influenced by the knowledge of that diathesis, in his treatment and prognosis. To the physician of experience, gout, whether existing as a local affection of the joints or as a pulmonary or cardiac disease is nevertheless gout, and requires treatment in accordance with that idea.

*A Thesis recommended for publication by the Faculty of the Buffalo Medical College.

Trousseau* has admirably expressed this sentiment in regard to syphilis: he says--"The special tendency engendered by the introduction of the syphilitic virus into the ecomomy, shows itself by very different lesions, affecting different tissues; but these lesions, however different they may be in appearance, all arise from the same cause, and are, in reality, all of the same nature, * * * * * * * To the real physician, exostosis, alopecia, psóriasis, roseola, bubo and chancre, are always syphilis—syphilis in different garbs, * * * * * * * I have spoken of the common manifestations of this disease; but under how many other forms of which we cannot always appreciate the nature, is it not concealed! How many nervous symptoms which appear as its sole phenomenal expression are dependent upon it, symptoms which remain inexplicable till the more decided characters of the diathesis which has produced them, afford the key to the diagnosis."

That most medical men are aware of the fact that syphilis may attack the nervous system at some time during the course of the disease, is doubtless true; that its possible connection with certain inexplicable nervous phenomena, is often overlooked seems none the less certain. Perhaps the cause of this may lie in the fact, that nervous affections dependent upon syphilitic origin, do not in many of their manifestations, differ materially from those in the non-syphilitic, and that at the time of their appearance, the other manifestations of the syphilitic virus are so unimportant and in many instances so masked, that they are overlooked in the anxiety caused by the more grave lesion. This does not however always obtain, as cases have been observed in the early stages of the disease when the other symptoms were quite prominent.

Having, through the kindness of Prof. J. F. Miner, had my attention called during the past year to several instances in which nervous affections were directly traceable to syphilis, and in which the administration of anti-syphilitic remedies was followed by marked improvement, I considered the subject of sufficient importance to form the basis of an inaugural thesis. It is unnecessary to say that it is not the object of this paper to advance any

* Clinical Medicine. Philadelphia, 1873. Vol. II, pp 601-602.

new theories concerning syphilis of the nervous system or its treatment. One or two points may be mentioned which have not fallen under my observation in a limited course of reading upon the subject; to present them as original would, however, be jumping at a conclusion which a limited acquaintance with authorities may render unjustifiable.

The history of syphilitic affections of the nervous system, extends as far back almost as the history of the malady. Recognized early, they have received the attention of numerous writers down to the present time. Special monographs upon the subject have been written, and chapters devoted to its consideration, have been incorporated in the more valuable and complete treatises upon venereal diseases, while the pages of various medical periodicals have from time to time contained communications upon the subject. It is during the last ten or fifteen years, however, that the most valuable additions have been made to our knowledge of the subject.

Syphilis may affect the nervous system in three ways:

I. Without any change in the nerve tissue itself.

(a.) By pressure from growths in neighboring parts, as exostoses, gummy tumors, etc., etc.

(b.) By changes in the nerve tissue, but not of syphilitic character, as softening due to pressure or to the obliteration of an artery by syphilitic disease.

II. The nerve tissue itself is affected.

III. Certain cases are observed in which on *post mortem*, no changes can be found in the nervous system, but evidence of syphilitic diseases can be found in other organs.

The phenomena observed in the last variety are very interesting, but are not fully explained. They offer therefore a promising field for investigation. I shall endeavor in the course of this paper to give my own explanation of this cause in certain instances. The classification above is a slight modification of that of Hill. *

*Affections of the Membranes of the Brain.*

Commencing with the membranes of the brain, I shall endeavor

* Syphilis and Local contagious Diseases. By Berkeley Hill, M. B., F.R.S. Philadelphia, 1868.

briefly to consider the different modes in which syphilis may affect the nervous system, their diagnosis, prognosis and treatment.

Of the coverings of the brain, that most frequently affected, is the dura mater. The affections of the other membranes, so closely simulate those of the dura mater, that it is unnecessary to consider them in detail. What is said of one may be taken as applying to all. The inflammatory changes in the dura mater are diffused, or circumscribed in the form of gummy deposits. Syphilitic pachymeningitis may be external or internal.

External pachymeningitis is most frequently accompanied by, or is the sequel of, osseous lesions. The internal tables of the skull being affected by caries or necrosis, the disease extends itself by continuity of structure to the dura mater producing inflammatory action. In these instances the dura mater is sometimes separated for some space from the bone, the interval being occupied by the products of the inflammatory process; or it may form adhesions to the cranium, of an exceedingly intimate nature, frequently involving the other membranes and the brain also. In either case, the nervous system will be more or less affected either by the pressure caused by the deposit or by the general inflammatory disturbance. Internal pachymeningitis so closely coincides with affections of the meninges that it is extremely difficult to diagnose the exact limit of the disease; in fact it seems hardly probably that the inflammatory action is present in one without extending itself to the others. Cases may, however, be observed in which the lesion is confined to one structure, in which the thickening and fibrinous appearance, the yellowish exudation, or the material constituting the gummy deposit, originates in, and is confined to one membrane. These cases must be rare however, and their exact diagnosis in life is impossible.

Gummy deposits in the membranes of the brain, by their pressure, often give rise to most serious symptoms. These tumors if not arrested in their growth, may involve the brain substance, or by their pressure give rise to local softening. Lancereaux* mentions a case of Sansons, in which a tumor situated in the anterior portion of the left hemisphere was continuous with the dura ma-

*A Treatise on Syphilis, Historical and Practical. By Dr. E. Lancereaux. Vol. II. P. [illegible]

ter by its external surface, and with the white substance of the brain by its internal surface. These tumors may also cause obliteration of the cerebral arteries, producing cerebral anæmia and softening. Cases have been observed in which the basilar, carotid middle cerebral, and other important arteries have thus been obliterated. The cranial nerves may also be pressed upon at their points of exit from the cranium.

SYMPTOMS. The symptoms observed in syphilitic pachymeningitis do not in most instances differ from those occurring from other causes. In the cases implicating the cerebral dura mater, vertigo, headache, epileptiform convulsions,* and rarely hemiplegia are the prominent features. When the coverings of the cerebellum are attacked, nausea, vomiting and photophobia are added to the other symptoms. Disturbance of the intellectual faculties is more common than paralysis. Localized pain, and paralysis of a single muscle or set of muscles, would indicate the probable presence of a gummy tumor.

DIAGNOSIS. The differential diagnosis between syphilitic and non-syphilitic meningitis is not always an easy task. The symptoms in syphilitic meningitis, (by the term meningitis is implied inflammation of the dura mater as well as the meninges,) are developed slowly; the patient complaining for some time of slight headache, dizziness, etc., with perhaps loss of memory. Should meningitis occur in a patient known to be syphilitic, or should he present that peculiar cachexia observed in cases of this character, syphilis may be suspected. As an aid to diagnosis it should be remembered that syphilitic diseases of the nervous system often assume peculiar characteristics, and should a case present itself in which the usual symptoms are accompanied by strange and unusual phenomena, a syphilitic cause should always be looked for.

PROGNOSIS. The prognosis is of course much influenced by the part implicated. It is however not unfavorable as, in most instances, the nerve substance is but little implicated. Recovery may be looked for, when, in meningitis from another cause, a fatal result would be expected.

---

* Syphilitic Epilepsy. By Reuben A. Vance, M. D. American Journal of Syphilography and Dermatology, July, 1871.

Treatment. The treatment in these instances is essentially the treatment of the cause. Bromide of potassium may be administered with a view of decreasing the amount of blood sent to the brain, but the chief reliance must be placed upon the iodide of potassium either alone or in combination with mercury. Hammond recommends ten to twenty grains of the iodide in combination with one sixteenth of a grain of bi-chloride of mercury, three times daily. These cases must not be treated by active antiphlogistic measures, on the contrary, a supporting (not stimulating) diet should be ordered in liberal allowance. The practice which is followed by some of treating similar cases by ice bags, blood letting, and active purgation would doubtless in these cases tend to aggravate rather than ameliorate the condition.

### *Affections of the Brain.*

Syphilitic affections of the brain are not so common as those of the membranes. When the brain is affected, the disease in the majority of instances, does not originate in it, but extends to it from contiguous structures. The brain may undergo, through the influence of syphilis, changes which manifest themselves in induration in some instances, in others, in softening; but the most frequent pathological change, and perhaps that most characteristic of syphilis, is the gummy tumor or nodule.

Gummy tumors are generally multiple and occupy various positions in the hemispheres of the brain, their most frequent seat is the vascular localities. Formed from the interstitial cellular tissue of the brain, they often are similar in shape to the portion of the brain in which they are situated. They are surrounded frequently by a zone of fibrous material, from which they can be readily enucleated. Gummy tumors of the brain vary in size and number, sometimes being as small as a pea, and occurring collectively, at others they attain the size of a filbert or walnut and may occur singly. In appearance, these tumors are whitish or yellowish, having a firm cartilaginous consistence, or are soft and caseous. They do not differ from the gummy deposits in the liver and other organs, and like them undergo a fatty metamorphosis by which they lose their consistence, become soft and yellowish, and if not surrounded by a fibrous sheath or zone become undistinguishable from the brain substance on simple inspection. In some cases they become absorbed, leaving in their places empty cyst, or cicatrices, which accounts in a measure for the cicatricial appearances or cystic tumors which have been found in the brain. Cases have been observed and placed upon record,* in which the whole of one hemisphere had become one large cyst. Gummy tumors of the brain should not be confounded with tubercle or with fibrous or cancerous growths, nor their remains with old serous or apoplectic cysts. They have less vascularity than fibrous or cancerous tumors, but in this respect and a few others, resemble tubercle. Tubercle does not however present so clear a separation from the brain, the fatty degeneration is more uniform, while in the gummy tumor it commences at the centre. Apoplectic cysts are more rounded than the remains of gummy tumors, and their walls are stained by the coloring matter of the blood. The changes in the arteries would distinguish the remains of an apoplectic clot from the cicatrix of a gummy tumor.

Symptoms. A tumor of considerable size may exist in the brain for an indefinite period, and produce no disturbance until a short time before death, or it may remain wholly unrecognized by any disturbance which it produces, and its discovery after death, be the result of accident. Pain, is in most cases the first symptom, but is not of course in itself diagnostic. It may be confined to the locality of the tumor or may be seated in another portion of the head. Disorders of the special senses and paralyzation of a single muscle or set of muscles, is a frequent symptom. Vertigo often to an extreme degree, and in some instances, vomiting are met with. Convulsions, either general, or convulsive movements of the limbs, are prominent features of the disease. The mental faculties are not affected as a rule until near the termination of the disease. Although headache is not in itself diagnostic of tumor of the brain, yet in no malady is it probably so severe and persistent.

Diagnosis. Although I should hesitate to subscribe to the assertion, that the diagnosis of cerebral tumors, is, with few exceptions rather a guess than a diagnosis; yet it seems to me that in

* Dr. Meyer. Dr. Calmeil.

many instances the difficulties to be met with, would be very great. All the above mentioned symptoms are to be taken into account in each case under consideration. Paralysis of parts supplied by the cerebral nerves, is a diagnostic sign of great value. These paralyses are, as a rule, peripheral when due to a tumor, cerebral when from other causes. (The distinction between the two is to be made by the application of the induced current to the nerve, in central paralysis it contracts normally, in peripheral it does not contract.) The seat of the tumor may be conjectured by observing the nerves affected. The syphilitic origin is to be diagnosed by the history, present symptoms, etc, etc.

Prognosis. The prognosis in cerebral tumors is generally that of fatal result, but a better result may be looked for when syphilitic tumors are suspected.

Treatment. This consists in the prompt and free administration of anti-syphilitic agents. Anodynes should be given to allay pain. Tonics and good food if needed.

Under the influence of syphilis the brain may become as tough as when steeped in alcohol. The induration is not always, alone found, but softening may exist with it. The symptoms observed as such as are seen in induration, or softening from other causes, often accompanied as in other syphilitic brain affections by peculiar inexplicable symptoms. The prognosis is to depend on the severity of the symptoms, but an unfavorable prognosis should not be given too early, as patients frequently recover a fair condition of health.

The nutrition of the brain is sometimes affected by syphilis, and we have either a condition of cerebral anæmia or hyperæmia.

When we remember that anything that will retard the free access of blood to the brain, will cause cerebral anæmia, we can readily imagine that syphilis may act as an agent in producing that condition. Niemeyer* gives as a cause of cerebral anæmia, any agent which produce a diminution of the space within the skull, as a tumor, thickening of the membranes or of the cranial bones.

Perhaps the most frequent cause of anæmia, is disease of the

*A Text Book of Practical Medicine, Vol. II. Pg. 171.

circulatory apparatus. The circulation may be cut off by the pressure of a cerebral tumor. Virchow, Lancereaux, Hughlings Jackson and others, have observed instances in which the circulation has thus been interfered with. The arteries are also subject to syphilitic disease which diminishes their calibre. Dr. Moxon reported a case * observed by him, in which the basilar artery had its channel decreased to about half its size, by the deposition of lymph between the coats of the vessel; other of the cerebral arteries were similarly affected, and the brain substance in certain localities softened. These changes were of undoubted syphilitic origin. Thrombosis and embolism from syphilis are probably rare. The former may result from syphilitic disease of the arteries and remain stationary, or becoming detached be carried along giving rise to embolism. Gummy tumors have been known to break into the cavity of the heart† and their contents form the nucleus for an embolus. Space forbids a consideration of these affections to a further extent. It remains only to be said, that unless early detected, syphilitic anæmia proves fatal. When disease of the arteries is suspected early it may yield to appropriate treatment. The prognosis when thrombi or emboli are formed, is not more favorable than when they result from other causes.

As a condition, the opposite to that which we have just been considering, we have cerebral hyperæmia. The disease of the arteries of the brain, instead of resulting in thickening, may produce a diminution in the thickness of the arterial walls; a loss of contractility resulting, we have a condition of hyperæmia. Cerebral hyperæmia may also be a secondary condition, the result of another wholly different nervous lesion. Bernard has shown that irritation or division of the sympathetic will produce a condition of hyperæmia upon the corresponding side. Little as is known concerning the syphilitic affections of the sympathetic, Dr. Petrow ‡ has conclusively shown in a recent paper, that it is the seat of syphilitic changes, which are more marked in old cases, and which will account for the condition of cerebral congestion known to exist in certain cases. The arteries may reach such a

* London Lancet Sept 25th, 1869.
† Lancereaux, Treatise on Syphilis. Vol. 1. Pg. 390.
‡ Virchow s Archiv.—London Lancet, June, 1873.

condition of tenuity, that under the force of the circulation, their walls give way and cerebral hemorrhage results. The same treatment as would be followed in hyperæmia, in other cases would naturally suggest itself here as an adjunct to the anti-syphilitic treatment.

*Affections of the Cord.*

There remains to be considered syphilitic affections of the spinal cord and of the nerves. Space will not permit the treatment which they deserve. I will therefore simply state the fact that the spinal cord and the membranes are subject to the same diseases which affect the brain, with however, less frequency. Exostosis and necrosis of the vertebral bones is also of less frequence than of the cranium, which may account to some degree for the difference in frequency.

The nerves are subject to pressure in their passage through osseous foramina, their sheaths are liable to syphilitic inflammation, and the nerve substance itself becomes changed and broken down. Gummy tumors have been found springing from the larger nerve trunks. These affections of single nerves, often give rise to local paralyses, which are inexplicable, until the influence of the syphilitic diathesis is considered. They yield, as a general rule, readily, to appropriate treatment. Often, the occurrence of neuralgia and rheumatism is explained, only when a syphilitic history is discovered.

Certain disorders of the urinary secretion, deserve here a passing notice. Prof. Jaksch has noticed that in certain instances diabetes accompanies syphilis, and attributes the reported cure of diabetes, by mercury, to a syphilitic origin of the disease. He thinks that the diabetes is due to an irritation of the floor of the fourth ventricle, producing it in a similar manner to that of Bernard in his experiments Mosler furnishes three cases in *Virchow's Archiv.* (1873) of diabetes dependent on neuropathic origin. One was syphilitic: post mortem showed softening in the left hemisphere, medulla and pons.

Conclusion. But little has been said in the preceeding pages concerning the time in which lesions of the nervous system are found. In the cases which I have had the privilege of observing, the period since the primary lesion, has varied from four years to eighteen. Nervous symptoms from syphilis may how ever arise in the early stages of the disease, even within a few weeks after the primary lesion. In the diagnosis of a syphilitic lesion of the nervous system, Dr. Keyes* regards, in hemiplegia especially, the fact that the attack occurs as a rule without loss of conciousness, a valuable aid to diagnosis. He also regards mydriasis, either alone or with other nervous symptoms, without positive disease of the eye, as presumptive evidence of syphilis.

Another valuable diagnostic sign, and one upon which I have endeavored to lay some stress, is the irregularity in the occurrence of the symptoms; they follow no regular order as is frequently observed in other instances.

In paraplegia, Keyes observes that it comes on slowly, (this has been the case in two cases now under observation) often without any local symptom to attract the attention to the injured part of the cord. The bladder almost always suffer more or less.

Certain cases are observed where no appreciable lesion can be found after death; paraplegia, hemiplegia, etc., having been observed in life; Dr. Keyes thinks that cerebral congestion will account for some of these cases. This is doubtless true, but the idea has occurred to me, and I find no author mentioning it, that reflex action may also account for a certain proportion, the irritation proceeding as in the theory advanced by Brown Sequard, from some of the internal organs, as the liver etc., or from the mucous membrane and skin.

Syphilitic epilepsy occurs late in life, and in patients who have not had attacks early in life. Many of the convulsive diseases of infancy and childhood, as also the cases of paraplegia met with at that period, are due to inherited syphilis.

The prognosis of syphilitic affections of the nervous system is better than for similar lesions, or even those less in extent dependent upon other causes. The prognosis should not however be expressed too favorably, for after the arrest of the disease, there will still often remain an inpairment of the nervous system which cannot be overcome: this will probably be slight, but it still re-

* Syphilis of the Nervous System. By E. L. Keyes, M. D. N. Y. Med. Jour. Nov. 1870.

mains as an enduring reminder of past affections of greater extent. I can still trace seven of the ten cases which I have observed; and in all there still remains a greater or less perversion of function: some of them are still under treatment, and still greater improvement may be looked for.

The most reliance in treatment is to be placed upon the iodide of potassium. Mercury may also prove a valuable aid: where for any reason its internal administration is undesirable, the oleate of mercury will be found to be more efficient than other forms of ointment; and more desirable for general use than fumigation.

There exists in the minds of some physicians a singular hesitancy in prescribing large doses of the iodide. It should be rapidly pushed to the limit of toleration. I have seen no more disturbance from one hundred grains three times daily than I have from twenty. It is but seldom that so large doses will be required however. Albuminuria occurring during the administration of iodide of potassium, as has been observed, countra-indicates its continuance.

I append the report of some of the cases which have fallen under my observation, hoping that a brief reference to some of their more distinctive features may be of interest.

(*To be Continued.*)

BUFFALO, Jan., 1874.

